# Environmental Asbestos Exposure as a Possible Pathway to Early‐Onset Pleural Mesothelioma: A Case From Afghanistan and Pakistan to London

**DOI:** 10.1002/rcr2.70774

**Published:** 2026-09-30

**Authors:** Sophie Mounouchos, Asmita Aggarwal, Asaad Ahmed Nafees, Emma Johnson, Paul Cullinan, Carl Reynolds

**Affiliations:** ^1^ Homerton NHS Healthcare Trust London UK; ^2^ University College London, Respiratory Medicine London UK; ^3^ Department of Community Health Sciences Aga Khan University Karachi Pakistan; ^4^ Imperial College London London UK; ^5^ London Centre for Work and Health London UK

**Keywords:** asbestos, environmental exposure, malignant mesothelioma, pleural

## Abstract

Malignant pleural mesothelioma (MPM) is strongly associated with occupational asbestos exposure and typically presents decades after exposure, in older men. The association of MPM with environmental exposure and genetic susceptibility is less recognised. We report a case of a 40‐year‐old man presenting in London with dyspnoea, weight loss and a persistent right‐sided pleural effusion following hospitalisation for pneumonia. Imaging showed circumferential nodular thickening of the right pleura with lymph node involvement, and histopathology was in keeping with epithelioid‐type MPM. With no known occupational exposure, childhood residences in Kabul, Afghanistan and Peshawar, Pakistan, where high levels of environmental asbestos are documented, represent plausible sources of environmental exposure. As a result of migration from regions with high environmental asbestos exposure, clinicians in high‐income countries may encounter increasing numbers of patients with MPM due to non‐occupational asbestos exposure.

## Graphical abstract

We report a case of a 40‐year‐old male with malignant pleural mesothelioma with no prior occupational exposure to asbestos. Environmental exposure as a cause of MPM may come into focus through migration from high environmental asbestos exposure regions in patients with no known occupational exposure.

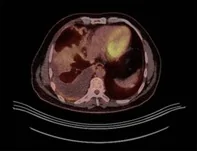

## 1 Introduction

Malignant pleural mesothelioma (MPM) is strongly associated with occupational asbestos exposure, predominantly affecting men in their 70s or older, with a typical latency of 30 years or more. The prognosis is poor with an average five‐year survival from diagnosis of 5%–10% [1]. Environmental (non‐occupational) asbestos exposure is an established but less common cause of mesothelioma [2]. Early‐onset mesothelioma is more frequently associated with environmental exposure and/or inherited susceptibility [3]. We report a case of pleural mesothelioma in a 40‐year‐old with suspected childhood environmental asbestos exposure in both Kabul, Afghanistan and Peshawar, Pakistan.

## 2 Case Report

A 40‐year‐old male was referred to a respiratory outpatient clinic in London due to persistent shortness of breath on climbing stairs and unintentional weight loss in the context of a recent hospital admission with severe community acquired pneumonia. His past medical history consisted of hypertension and bilateral renal calculi. He was an ex‐smoker with a five pack‐year history, having quit 8 years prior to presentation.

Follow‐up chest radiographs arranged to ensure resolution of his pneumonia showed a persistent right‐sided pleural effusion with overlying atelectasis (Figures 1 and 2). Computed tomography imaging demonstrated a moderate right‐sided pleural effusion with circumferential nodular thickening of the right pleura, enlarged precardiac lymph nodes and prominent right hilar lymph nodes. Subsequent fluorodeoxyglucose‐positron emission tomography (FDG‐PET) showed diffuse pleural uptake with moderate avidity and uptake in the diaphragmatic and right retrocrural lymph nodes (Figure 3). It also identified an avid intercostal lymph node, amenable to biopsy. Formal staging was T3N1M0.

**FIGURE 1:**
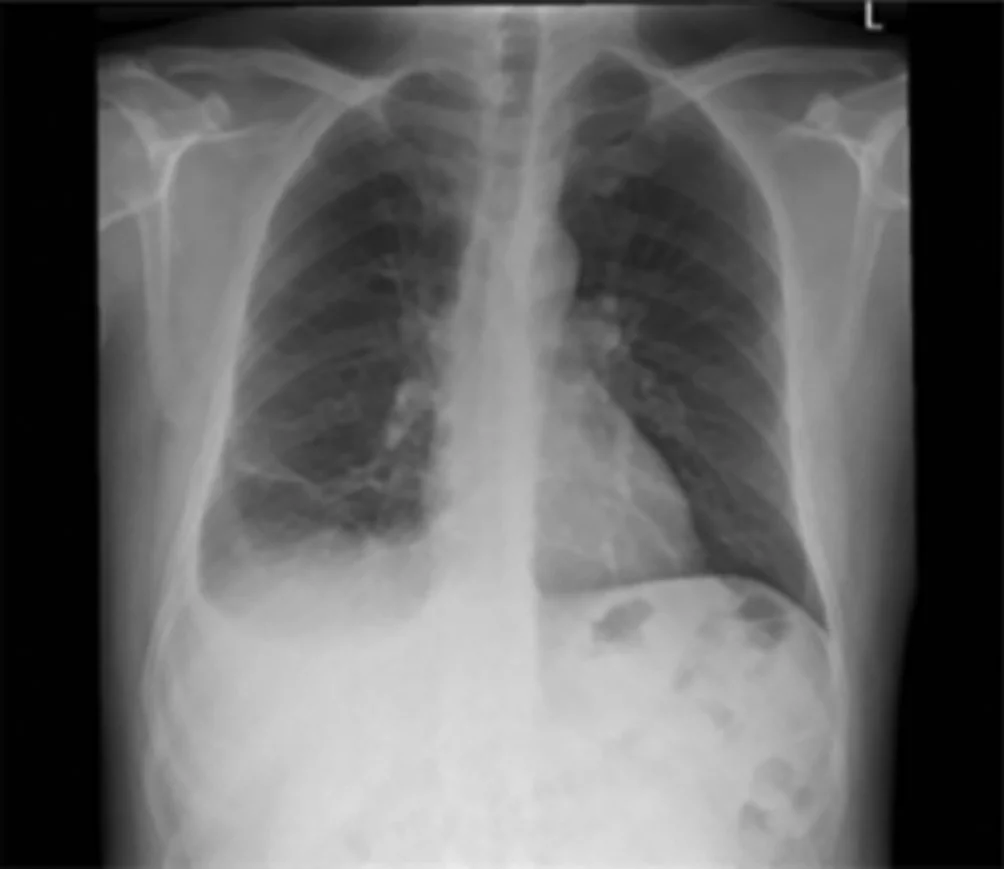
Right‐sided pleural effusion six weeks post‐treatment for pneumonia.

**FIGURE 2:**
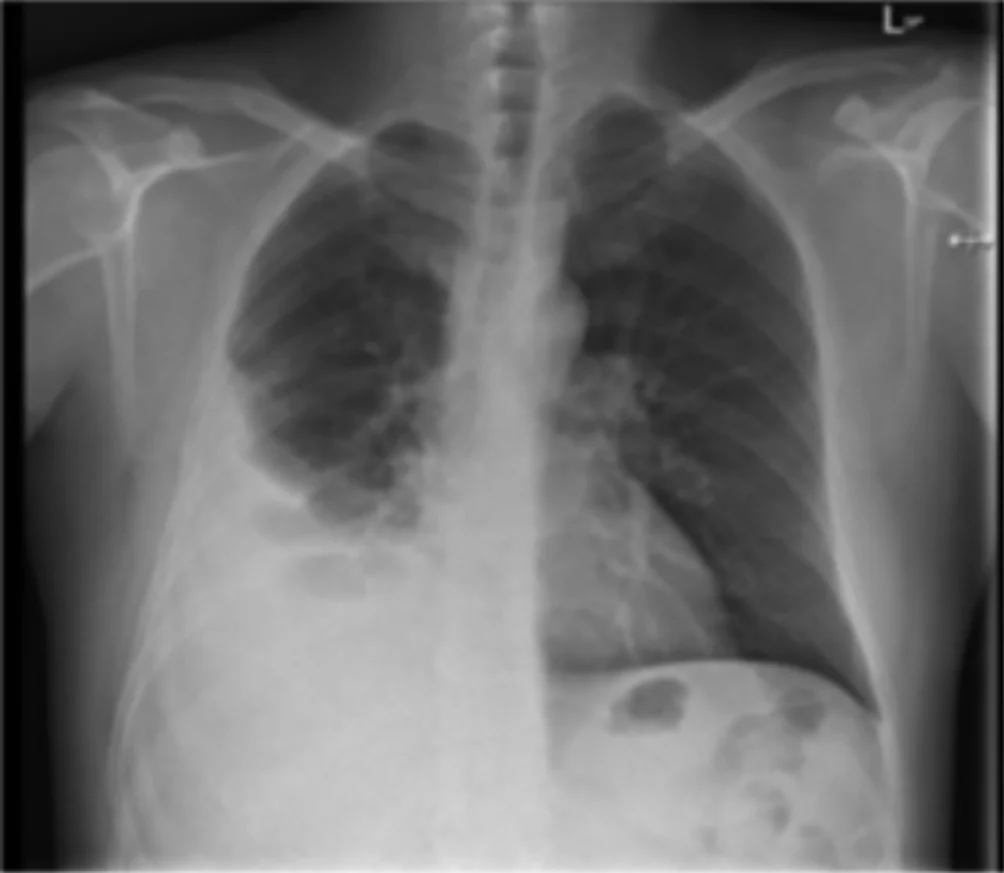
Persistent and enlarging right‐sided pleural effusion 10 months post repeat x‐ray.

**FIGURE 3:**
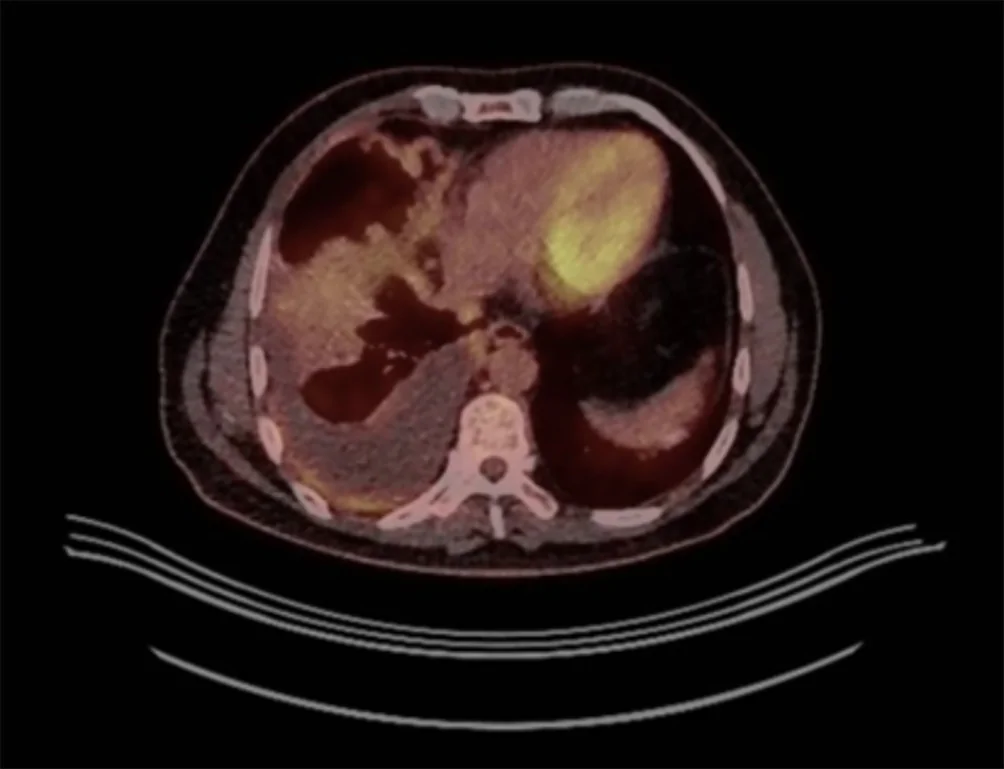
PET CT showing increased avidity in the right pleura and a right‐sided pleural effusion.

On thoracic ultrasound there was a simple right‐sided effusion with no septations. Cloudy yellow fluid was aspirated and analysis was consistent with an exudate; its protein level was 54 g/L with a fluid: serum protein ratio of 0.72, glucose 3.8 mmol/L and lactate dehydrogenase 490 units/L. Cytology showed lymphocytosis, with no malignant cells. Histopathology results from the avid intercostal lymph node demonstrated morphology and immunoprofile in keeping with an epithelioid‐type malignant mesothelioma with lymphovascular invasion, and negative adenocarcinoma markers. Tumour cells were positive for expression of Calretinin, WT‐1 and D2‐40 and negative for TTF‐1. Immunohistochemistry demonstrated retained BRCA‐1 associated protein (BAP1) expression. Germline testing for BAP1 tumour predisposition syndrome was negative; broader germline panel testing was not performed.

The case was discussed by the lung cancer multidisciplinary team, followed by referral to the regional mesothelioma team. The tumour was deemed inoperable and immunotherapy treatment with Nivolumab + Ipilimumab was started.

Social history revealed the patient was born in Kandahar, Afghanistan. At age 3 he moved to Kabul, later moving to Peshawar, Pakistan at age 13, before immigrating to the United Kingdom (UK) at age 16. He had no identifiable occupational history of exposure to asbestos, nor para‐occupational exposure from household contacts. He did not recall ever living near an asbestos mine. In the United Kingdom he worked as a hospital biochemist.

## 3 Discussion

In the United Kingdom, the decline of heavy industry together with progressive exposure legislation has resulted in falling occupational mesothelioma deaths, bringing potential environmental exposures into focus. In keeping with men having greater occupational exposure to asbestos, they experience the bulk of disease when all ages are considered. However, for those under 40 years of age, the distribution of mesothelioma across the genders is more even, suggesting other causal factors are important [3].

The environmental mesothelioma outbreak in Turkey linked with exposure to the asbestiform mineral, erionite, identified familial clustering and led indirectly to the identification of the BAP1 associated tumour predisposition syndrome [4, 5]. The significance of genetic predisposition was highlighted also in a United States‐based cohort of 198 patients with mesothelioma, where 12% had susceptible germline mutations, notably in younger patients [6]. As BAP1 accounted for only a quarter of the pathogenic germline mutations identified across 13 cancer‐susceptibility genes in the US cohort [6], inherited susceptibility cannot be fully excluded in our patient. These factors point to a distinct entity of mesothelioma in the young, with a different natural history from traditional MPM of the older patient with occupational exposure.

In search of potential sources of asbestos exposure for our patient, we identified the growing body of research on MPM and pleural plaque cases in Afghanistan and Pakistan associated, it seems, with environmental exposure. In Afghanistan, pleural plaques have been reported in a 51‐year‐old man who resided in Kabul with no occupational asbestos exposure. The authors proposed environmental exposure from the neighbouring Loghar region, where natural deposits of tremolite, an amphibole asbestos, have been documented [7]. Additionally, asbestos‐containing materials, including asbestos‐cement piping and sheeting, were used in Kabul during the 1970s [8, 9]. During the 1980s, the city was subjected to repeated structural damage during the Soviet‐Afghan conflict. Armed conflict may have mechanically disrupted such materials, generating airborne asbestos fibres; although the importance of this as a mechanism is uncertain.

Subsequent residence in Pakistan, where asbestos‐containing construction materials remain widely used, may have contributed additional low‐level environmental exposure. Peshawar lies within the Khyber Pakhtunkhwa province, in which 90% of Pakistan's naturally occurring asbestos deposits exist [10]. A case series identified a heavy burden of malignant mesothelioma, thought to be associated with the high concentration of asbestos deposits in the region [11]. Concern regarding rising diagnoses in younger individuals is reinforced by a recent case‐report in Northern Pakistan; in 2011 a 26‐year‐old female was diagnosed with MPM, with no previous occupational exposure to asbestos [12]. The interval between early childhood residence in Kabul and Peshawar, and diagnosis at age 40 is consistent with typical recognised mesothelioma latency periods.

Clinicians in high‐income countries should consider earlier life environmental and residential exposures acquired overseas, particularly in younger patients with mesothelioma who lack conventional occupational exposure histories. Genetic susceptibility and its interaction with the environment may be of greater importance in younger patients without occupational exposure. Migration to high‐resource healthcare settings may represent an important mechanism for recognising environmental mesothelioma in lower‐resource settings.

## Author Contributions

**Sophie Mounouchos:** conceptualization, writing – original draft, writing – review and editing, visualization, project administration. **Asmita Aggarwal:** conceptualization, writing – original draft, writing – review and editing, visualization. **Asaad Ahmed Nafees:** conceptualization, writing – review and editing, validation. **Emma Johnson:** writing – review and editing, conceptualization, writing – original draft. **Paul Cullinan:** writing – review and editing, supervision, writing – original draft, conceptualization, resources. **Carl Reynolds:** conceptualization, methodology, data curation, supervision, writing – review and editing, formal analysis, investigation, writing – original draft.

## Funding

The authors have nothing to report.

## Consent

The authors declare that written informed consent was obtained for the publication of this manuscript and accompanying images and attest that the form used to obtain consent from the patient complies with the Journal requirements as outlined in the author guidelines.

## Conflicts of Interest

The authors declare no conflicts of interest.

## Data Availability

Research data are not shared.
